# Behaviour-based functional and dysfunctional strategies of medical students to cope with burnout

**DOI:** 10.1080/10872981.2018.1535738

**Published:** 2018-10-29

**Authors:** Rebecca Erschens, Teresa Loda, Anne Herrmann-Werner, Katharina Eva Keifenheim, Felicitas Stuber, Christoph Nikendei, Stephan Zipfel, Florian Junne

**Affiliations:** a Department of Psychosomatic Medicine and Psychotherapy, Medical University Hospital Tuebingen, Tübingen, Germany; b Department of General Internal Medicine and Psychosomatics, University Medical Hospital Heidelberg, Heidelberg, Germany; c Deanery of Students’ Affairs, University’s Faculty of Medicine, Tübingen, Germany

**Keywords:** Medical students, stress, burnout, exhaustion, cynicism, academic efficacy, MBI-SS, reference population

## Abstract

**Background**: High levels of burnout rates amongst medical students have been confirmed by numerous studies from diverse contexts. This study aims to explore the functional and dysfunctional coping strategies of medical students with regard to their respective burnout factors.

**Methods**: About 845 medical students in the 3^rd^, 6^th^, and 9^th^ semesters and students in their final year were invited to take part in the survey. The self-administered questionnaire included items on potential functional and dysfunctional behavioural-based coping strategies as well as the Maslach Burnout Inventory-Student Version (MBI-SS). In addition, a comparison of the local results with a German reference sample involving other students was calculated.

**Results**: A total of 597 medical students (70.7%) participated in the cross-sectional study. The results showed high burnout rates, averaging 35%. Students in earlier stages of university education showed lower values for cynicism (a subdimension of burnout), but higher values for emotional exhaustion than students in higher stages. Concerning academic efficacy, there was a trend towards less efficient perception among students in higher education. The identified functional coping strategies were ‘*seeking support from friends*’, ‘*seeking support from family*’, ‘*doing relaxing exercise*’, ‘*doing sports*’ and ‘*seeking support from fellow students*’. The identified dysfunctional coping strategies were ‘*taking tranquilizers*’, ‘*taking stimulants*’, ‘*drinking alcohol*’, ‘*withdrawal and ruminating*’, and ‘*playing games on the PC or mobile phone*’. The medical students surveyed are more affected by burnout symptoms than the reference populations, but the overall result was difficult to interpret.

**Conclusions**: The identified behavioural-based functional coping strategies suggest that social support and active relaxing exercises seem to be very important possibilities for medical students to reduce stress and exhaustion. The use of drugs and alcohol for stress reduction raises concerns. Programs are recommended to improve resilient behaviour and to impart the identified functional coping strategies to medical students.

## INTRODUCTION

The investigation of mental health of medical students began in the early 1980s with the introduction of the Burnout Concept and the Maslach Burnout Inventory by Christina Maslach [1–3]. Burnout can be characterized by a ‘core dimension’ that includes physical and mental exhaustion [4,5]. In addition, burnout may be associated with a sense of emotional distance to the curriculum or to patients in the sense of increased ‘cynicism’ [6–8]. Furthermore, feelings of ‘low academic efficacy’ may occur when students perceive themselves as less competent and successful [9,8].

Many studies have investigated stress and burnout in medical students in different study designs, with different assessment instruments and with associations, for example, on gender, stage of medical education and specific stressors [10–14]. In a systemic review with meta-analysis, Erschens and colleagues [14] identified the Maslach Burnout Inventory (MBI) as one the most common and effective instrument for measuring burnout. They found prevalence rates of relevant burnout of 7.5%–75.2% among medical students, measured with MBI Student Survey (MBI-SS). In addition to the knowledge of prevalence rates and specific stressors, it is also necessary to investigate coping and resilience behaviour associated with burnout in different stages of university education. These findings can then be used collectively to develop prevention and intervention programs for medical students at the behavioural level and provide implications for medical education on ‘how to stay healthy as a future physician’. According to Lazarus and Folkman, ‘coping can be understood as a constant cognitive and behavioural attempt to deal with specific external and internal demands that are perceived and valued as stressful’ [15,16]. The main aim of this work is to examine this relationship of coping behaviour and burnout, whereby the theories and concepts of Lazarus and Folkman [16] and Carver and colleagues [17,18], splitting coping strategies according to their functionality into functional and dysfunctional coping strategies, are applied to the context of medical students.

Erschens and colleagues found higher values for burnout in their meta-analysis among medical students in the included studies than in the age-appropriate standard population sample [14]. In an auxiliary question, this study also examines the question of whether medical students are also more exposed than students of other disciplines. There are still unclear findings here [19–21].

This study focused in particularly on the association of burnout among medical students in different stages of medical curriculum with a variety of possible functional and dysfunctional coping strategies. (I) It was investigated in detail how functional and dysfunctional behaviour-based coping strategies can reduce or increase the possibility of being burnt out in medical students. (II) In addition, the burnout results for medical students measured with the MBI-SS were compared with a reference sample of students from other disciplines [22].

## MATERIALS AND METHODS

### Design and procedure

#### Cross-sectional study

The study was conducted at a German medical faculty applying a cross-sectional study design. Results for global stress, measured with the Perceived Stress Questionnaire (PSQ-20) German Version with 20 items [23] in the same study cohort and their association to specific private- and training-related stressors, have been published elsewhere by Erschens and colleagues [24]. A total of 845 medical students were invited to participate in the study, which was conducted beginning with the winter semester in October up to the start of the summer semester in April. Burnout and coping strategies were measured in four groups of medical students in different stages of medical education.

#### Comparison with reference population

The burnout results for medical students of the cross sectional study were compared with a German Reference sample of students from other disciplines by Gumz and colleagues [22]. In their study, 377 students (68.4% women, *M* = 23.5 years, *SD* = 2.8 years) were interviewed with the primary aim of examining the factor structure and psychometric criteria of the German translation Maslach burnout inventory for students (MBI-SS) by Schaufeli and colleagues [8].

The students came from different fields of study such as social sciences, law, natural sciences and humanities and students of human medicine [22].

### Setting and participants of the cross-sectional study

#### Medical education in Germany

Medical Education in Germany takes 12 semesters or 6 years and is divided into a pre-clinical study part (semesters 1–4), a clinical study part (semesters 5–10) and a clinical-practical study part (semesters 11 and 12). The medical curriculum is structured by the German Medical Licensing Regulations (Ärztliche Approbationsordnung, ÄAppO) [25]. While in the pre-clinical part of the education the emphasis is on natural sciences, a dissection course and the first part of a course on medical conversation, the contents of the second part of the study are general clinical subjects such as internal medicine, general medicine or ophthalmology, cross-sectional subjects (e.g. radiology, pain medicine or pharmacotherapy), as well as weekly internships and practical exercises an the second part of communication courses. In the last (clinical-practical part), students need to demonstrate full-time clinical-practical work of a total of 48 weeks in three disciplines (internal medicine, surgery and general medicine or another clinical-practical elective). There are a total of three state exams (known as M1–M3). The first section of the Medical Examination (M1) is both written and oral and can be completed after 4 semesters of preclinical time. The second part of the medical examination M2 is conducted in writing after the clinical and before the clinical-practical period and the third part of the medical examination is conducted orally after the 12th semester, after completion of the clinical-practical period. For an overview of medical education in Germany see Nikendei and colleagues [26].

#### Participants of the cross-sectional study

For comprehensive information on different burnout rates and coping strategies, it was important to survey students from the pre-clinical, clinical and clinical-practical study phases. Medical students in the 3^rd^, 6^th^ and 9^th^ semesters were invited to voluntarily participate in the study in communication and psychosomatic courses after the class was over. The courses were easily accessible as they were taught in the Department of Psychosomatic Medicine and Psychotherapy by colleagues of the authors. The students were able to deny or terminate the survey at any time and without any disadvantage. The survey was completely anonymous and in paper-pencil form. The survey was conducted throughout the semester because the students were taught in small groups. In order to avoid burnout peaks, no semesters were surveyed that were close to the three state exams (i.e., semesters 4, 10 and 12). Final year students were interviewed under the same conditions as described above with the assumption that they were interviewed with an online version of the questionnaire, since local accessibility was very difficult.

#### Responder and non-responder

At the beginning of the survey, a questionnaire was placed at each place where a student was sitting (for the final year students we sent the email with the invitation to all students over two trimesters via the Dean’s Office). Students who answered the questionnaire and submitted it to an anonymous urn were counted as responders (final year students were counted as responders who submitted the questionnaire with a coded IP address). Students who leave the questionnaire unanswered, who only read the questionnaire out of interest, who finished the questionnaire after a few pages, counted as non-responder (final year students were counted as non-responders who not answer to the survey).

### Ethics statement

The Ethics Committees of the Faculty of Medicine at University Hospital Tübingen approved the study (number 053/2014BO1). All medical students provided their written informed consent.

### Measurements

The scope of the questionnaire included demographic information such as age and gender. Burnout among medical students was measured by the Maslach Burnout Inventory–Student Survey [8] with the German version of the validation study by Gumz and colleagues [22]. Behavioural-based coping strategies were evaluated on the basis of a broad spectrum of newly designed items based on qualitative material, which is explained below.

### The Maslach burnout inventory–student survey

The Maslach Burnout Inventory–Student Survey (MBI-SS) consists of 15 items^1^ that constitute three scales: emotional *exhaustion* (5 items), *cynicism* (4 items), and *academic efficacy* (6 items). All items are scored on a 7-point frequency rating scale, ranging from 0 (never) to 6 (always). High scores on exhaustion and cynicism and low scores on academic efficacy are indicative of burnout [8]. Currently, there are no official cut-off criteria for the individual subscales and for the definition of burnout with respect to the MBI-SS [27]. Different authors use very heterogeneous cut-off criteria, which makes comparison difficult. According to the findings revealed by the review of Erschens and colleagues [14], the cut-offs for the Maslach Burnout Inventory-Human Services Survey (MBI-HSS) were converted and transferred to the three subscales of the MBI-SS with regard to the official burnout manual of Maslach [1,4]. Thus ‘high exhaustion’ (EE) was defined with the sum values ≥16, ‘high’ cynicism (CY) rates with the sum values ≥10 and ‘perceived low level of academic efficacy’ (AE) with the sum values <24. Cronbach’s alpha coefficients ranged from α = .81 to α = .86 [22].

For the present cross-sectional study, burnout was defined according to the more comprehensive approach, which is particularly applied to “healthy cohorts, for example, [28,29,27]. Thus, burnout is defined with high values for subscale exhaustion (score ≥16) or cynicism with a score ≥10. Accurately the mean values for all subscales EE, CY and AE are evaluated with SDs and prevalence rates for high values.

### Measurements of functional and dysfunctional coping strategies

**Figure 1.**
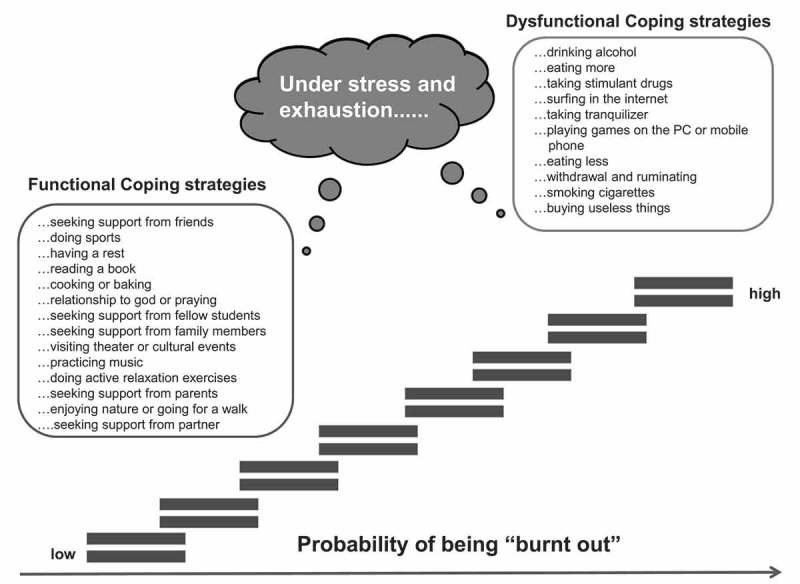
Illustration of hypothesised functional and dysfunctional coping strategies and their association with burnout. The use of functional coping strategies should be associated with a low probability of being burnt out. The use of dysfunctional coping strategies should be associated with a high probability of being burnt out.

According to their functionality, coping strategies were divided into functional and dysfunctional coping strategies [18,16]. The functional and dysfunctional coping strategies were developed on the basis of qualitative material. On the one hand, this includes the evaluation of written reflection work by medical students on possible stressors and resilience factors during university education [30] as well as existing qualitative studies [31,32], and the results of informal expert groups as well as a review from the literature [14]. Items were developed to identify and assess potential functional and dysfunctional coping strategies. Possible behaviour-based coping strategies were grouped either as functional coping strategies that reduce the probability of suffering from burnout, or as dysfunctional coping strategies that increase the probability of being affected by burnt-out symptoms. Both groups of strategies have been designed in a behavioural way to facilitate the development of specific prevention programs to improve the existing resilient behaviour and to establish programs to learn the identified strategies. The final instrument included a total of 25 functional and dysfunctional coping strategies. Students were asked how they generally act when they are stressed or exhausted. In a two-tailed design, the students rated *yes (yes, I use this coping strategy*) or *no (no, I do not use this coping strategy*) if the presented coping strategy was used. Secondly, medical students rated the strategies that they did use on a 5-point Likert scale with regard to their perceived effectiveness in preventing stress and exhaustion with 0 = ‘not at all satisfied; 1 = ’slightly satisfied”; 2 = ”moderately satisfied”; 3 = ”very satisfied” and 4 = ‘completely satisfied’. Figure 1 shows a detailed list of analysed functional and dysfunctional coping strategies. The students had the opportunity to indicate further coping strategies they used, which were not offered by the questionnaire, in free-text fields.

### Statistical analysis

Statistical analyses were performed with IBM SPSS for Windows version 23.0 and MATLAB. Group difference for all three dimensions – *emotional exhaustion, cynicism* and *academic efficacy* – plus existing burnout were examined for all subgroups. Due to the psychometric nature of the burnout data, nonparametric tests, such as the Kruskal–Wallis test and the Man–Whitney U test, were used.

Hypothesised coping strategies were tested with one-way chi-square tests and reported as odds ratios (ORs) with the corresponding confidence limits and p-values. For this purpose, we have subdivided the sample into ‘burnt out’ and ‘not burnt out’ with high values for subscale exhaustion (score ≥16) or cynicism with a score ≥10. According to our hypotheses, functional coping strategies should be associated negatively with burnout and dysfunctional coping strategies should be associated positively with burnout. The odds ratios are to be interpreted as meaning that when using a functional coping strategy, the probability of being without burnout is higher than when not using a functional coping strategy. And vice versa, when using a dysfunctional coping strategy, the probability of having burnout is higher than when not using the dysfunctional coping strategy. Forest plots were applied to illustrate the results the coping strategies using R [33]. In addition to the analysis of odds ratios, an explorative binary logistic regression was performed to assess potential influences of the respective coping strategies on burnout. A stepwise forward selection with an alpha level of 0.05 was employed. The coping strategies (dummy coded as 0 = not use; 1 = use) are analysed as potential multivariable determinants of burnout (dummy coded as 0 = no burnout; 1 = burnout). For the comparison of burnout results obtained by medical students with the reference sample of students from other disciplines [22], a Z-test was calculated. The effect sizes were calculated and interpreted according to Cohen [34].

## RESULTS

### Response rate and sample description

A total of 597 of the 845 (70.7%) recruited medical students participated in the cross-sectional study. The majority of the participants were female with *n* = 372 (62.3%). Their ages ranged from 16 years to 60 years with *M* = 25.6 (*SD *= 4.4). Table 1 shows details for the response rate and demographics for each subgroup.

**Table 1.** Characteristics of study population and subgroups.

	Total sample	3^rd^ semester	6^th^ semester	9^th^ semester	Final year students	*p*
***N*/*n***	*N* = 597	*n* = 143	*n* = 123	*n* = 154	*n* = 177	*p* < .01**
**Response rate**	70.7%	79.4%	79.4%	85.6%	58.1%	p < .01**
**gender**						
female	N = 372, 62.3%	n = 90, 62.9%:	n = 67, 54.5%	n = 98, 63.6%	n = 117, 66.1%	p > .05
male	N = 225, 37.7%	n = 53, 37.1%	n = 56, 45.5%	n = 56, 36.4%	n = 60, 33.9%	p > .05
**Age** (years)	M = 25.6, SD = 4.4	M = 22.28, SD = 3.57	M = 23.05, SD = 4.00	M = 26.42, SD = 3.92	M = 27.77, SD = 4.10	p < .01**
[Min-Max]	[16–60]	[16–38]	[21–52]	[21–47]	[23–60]	p < .01**

### Burnout in different stages of university education

Table 2 presents results for the burden of burnout with the three subscales of emotional exhaustion (EE), cynicism (CY) and academic efficacy (AE) measured with MBI-SS in the different groups. The results showed significant group differences with Kruskal–Wallis for all three dimensions: EE with χ^2^ = 9.45; *df* = 3; *p* < .05, CY with χ^2^ = 31.74; *df* = 3; *p* < .01 and AE with χ^2^ = 8.17; *df* = 3; *p* < .05. A table with *post hoc* tests with Mann-Whitney U for the subdimensions and subgroups is available in the Supplemental Material 1. Students in the 9^th^ semester and students in the final year showed lower scores of EE than students in the 3^rd^ semester. For the subscale CY, students in earlier stages of university education showed lower scores than students in the higher stages. Except for the comparison between students in the 9th semester and final year students, all comparisons were significant. Feelings of AE rated higher in the 9^th^ semester than in the 3^rd^ and the 6^th^ semester. There was no significant group effect for the parameter ‘burnout’, with χ^2^ = .686; *df* = 3; *p* > .05. The prevalence rate ranged from 31.7 % to 36.4%.

**Table 2.** Results for sub dimensions of the Maslach Burnout Inventory (MBI-SS).

Dimension	Means and frequencies	3^rd^ semester	6^th^ semester	9^th^ semester	final year students	Total sample	p^6^
**Emotional** **Exhaustion** **(EE)**	M_sum_^1^(SD)	13.79(5.82)	12.72(5.43)	12.19(5.61)	11.85(6.46)	12.50(5.76)	< .01**
	M_scal_^2^(SD)	2.75(1.16)	2.55(1.09)	2.44(1.22)	2.37(1.29)	2.52(1.15)	< .01**
	**high EE**^3^ n,%	n = 46,% = 32.2	n = 26,% = 21.1	n = 32,% = 20.8	n = 42,% = 23.7	n = 160,% = 23.5	< .01**
**Cynicism (CY)**	M_sum_(SD)	3.49(4.40)	4.60(4.69)	6.20(5.48)	6.25(6.05)	4.91(5.38)	< .01**
	M_scal_(SD)	0.872(1.10)	1.15(1.17)	1.55(1.37)	1.56(1.51)	1.13(1.31)	< .01**
	**high CY^4^** n,%	n = 11,% = 7.7	n = 24,% = 19.5	n = 36,% = 23.4	n = 43,% = 24.3	n = 121,% = 17.8	< .01**
**Academic Efficacy** **(AE)**	M_sum_(SD)	25.85(4.98)	25.51(5.45)	23.94(5.45)	24.58(7.08)	25.02(6.02)	< .01**
	M_scal_(SD)	4.31(0.831)	4.25(0.912)	3.99(1.02)	3.92(1.02)	4.17(1.00)	< .01**
	**Low AE^5^** n,%	*n* = 45,% = 31.5	*n* = 41,% = 33.3	*n* = 66, % = 42.9	*n* = 63,% = 35.6	*n* = 244,% = 35.8	< .01**
**Burnout^7^**	n,%	*n* = 49,% = 34.3	*n* = 39,% = 31.7	*n* = 56,% = 36.4	*n* = 62,% = 35.0	*n* = 222,% = 32.6	> .05

^1^Mean value of the respective sum score for EE, CY and AE; ^2^Mean value of the respective scale score for EE, CY and AE; ^3^EE sum scores ≥16; ^4^CY sum score ≥10; ^5^AE sum score <24; ^6^ p-value for group differences with Kruskal-Wallis over all four semester groups for the respective burnout dimension in each line; ^7^EE(score ≥16) **or** CY (score ≥10); ** highly significant group difference

### Comparison with reference sample

Table 3 shows the comparison of current results for burnout among the investigated medical students, with the reference sample from Gumz and colleagues [22]. For the dimensions EE and AE, the investigated medical students claimed to be more exhausted and feel less efficient. For the dimension CY, there were no differences between the investigated medical students and the students from the study by Gumz and colleagues. The effect sizes also for the significant differences are classified as ‘low’ with *d* = .17 for emotional exhaustion and *d* = .19 for academic efficacy.

**Table 3.** Results of the comparison between the investigated sample of medical students and a general student reference sample.

	Current study	Reference sample^1^			
	medical students	students			
Dimensions	M_scal_^3^(SD)	M_scal_(SD)	z	p	d^2^
Emotional Exhaustion (EE)	2.52(1.19))	2.31(1.19))	2.64	< .001*	.17
Depersonalisation (DP)	1.13(1.31))	1.31(1.34))	−.011	> .05	.00
Academic Efficacy (AE)	4.17(1.01)	3.95[1.07))	2.91	< .001*	.19

^1^[22], ^2^ effect size in accordance to cohen’s d; ^3^ Mean value of the respective scale score for EE, CY and AE; *significant

## Functional and dysfunctional coping strategies

### Important functional and dysfunctional coping strategies associate with burnout

**Figure 2.**
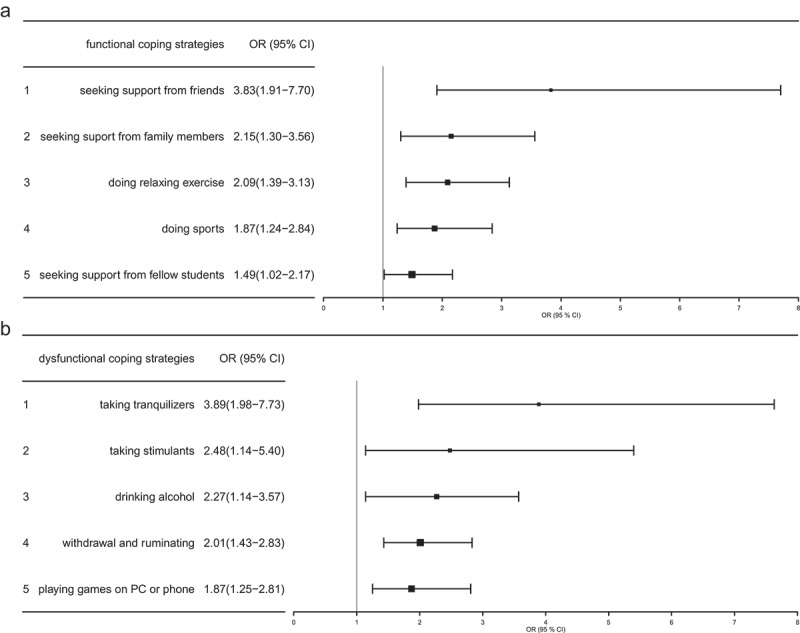
Illustration of hypothetised functional (Figure 2a) and dysfunctional (Figure 2b) coping strategies with associated odds ratios (OR). The horizontal lines represent the standard deviations and the squares are at the height of the x-axis and illustrate the respective value.

By calculating odds ratios, five relevant functional and dysfunctional coping strategies have been identified across all semester groups. Tables 4a and 4b show the results of this analysis. The use of the functional coping strategies *seeking support from friends (*OR = 3.83; 95% CI: 1.91 to 7.70; *p* < .01) *including family (*OR = 2.15; 95% CI: 1.30 to 3.56; *p* < .01), *doing relaxing exercise (*OR = 2.09; 95% CI: 1.39 to 3.13; *p* < .01), *doing sports (*OR = 1.87; 95% CI: 1.24 to 2.84; *p* < .01) and *including fellow students (*OR = 1.49; 95% CI: 1.02 to 2.17; *p* < .05) are negatively associated with burnout and reduce the probability of suffering from burnout, while dysfunctional coping strategies *taking tranquilizers (*OR = 3.89; 95% CI: 1.98 to 7.63; *p* < .01), *taking stimulants (*OR = 2.48; 95% CI: 1.14 to 5.40; *p* < .01), *drinking alcohol (*OR = 2.27; 95% CI: 1.14 to 3.57; *p* < .01), *ruminating (*OR = 2.01; 95% CI: 1.43 to 2.83; *p* < .01), and *playing games on the PC or mobile phone (*OR = 1.87; 95% CI: 1.25 to 2.81; *p* < .01) are positively associated with burnout and reduce the probability of suffering from burnout increase the probability of having burnout symptoms. Figure 2a and Figure 2b illustrates the comparison of functional and dysfunctional coping strategies using a forest plot.

**Table 4a.** Ranking of importance of investigated functional coping strategies.

Burnout								Total use
	Yes		No					
Relevant functional coping strategies^1^	***n***	**%**	***n***	**%**	χ^2^ value	*p* value	OR (95% CI]	*n*, %
**1. seeking support from friends**	182	32.5	378	67.5	16.09	<.01**	3.83 (1.91–7.70)	*n* = 560,% = 93.8
No use	24	64.9	13	35.1			1	
**2. seeking support from family**	171	32.6	357	67.6	7.47	<.01**	2.15 (1.30–3.56))	*n* = 528,% = 88.4
No use	35	50.7	34	49.3			1	
**3. doing relaxing exercise**	147	30.9	328	68.8	13.02	<.01**	2.09 (1.39–3.13)	*n* = 475,% = 79.6
No use	59	48.4	63	51.6			1	
**4. doing sports**	153	31.7	330	68.3	8.96	<.01**	1.87 (1.24–2.84)	*n* = 483,% = 80.9
No use	53	46.5	61	53.5			1	
**5. seeking support from fellow students**	142	32.1	300	67.9	4.264	<.05*	1.49 (1.02–2.17)	*n* = 442,% = 74.0
No use	64	41.3	91	58.7			1

**Table 4b.** Ranking of importance of investigated dysfunctional coping strategies.

Burnout								Total use
	Yes		No					
Relevant dysfunctional coping strategies^1^	***n***	**%**	***n***	**%**	χ^2^ value	*p* value	OR (95% CI)	
**1. Taking tranquilizers**	26	65.0	14	35.0	17.64	<.01**	3.89 (1.98–7.63)	*n* = 40,% = 6.7
No use	180	32.3	377	57.7			1	
**2. Taking stimulants**	15	55.6	12	44.4	5.54	<.05*	2.48 (1.14–5.40)	*n* = 27,% = 4.5
No use	191	33.5	379	66.5				
**3. Drinking alcohol**	46	51.1	44	48.9	12.93	<.01**	2.27 (1.14–3.57)	*n* = 90,% = 15.1
No use	160	31.6	347	68.4			1	
**4. withdrawal and ruminating**	123	42.6	166	57.4	16.09	<.01**	2.01 (1.43–2.83)	*n* = 289,% = 48.4
No use	83	26.9	225	73.1			1	
**5. playing games on PC or mobile phone**	56	46.3	65	53.7	9.31	<.01**	1.87 (1.25–2.81)	*n* = 121,% = 20.3
No use	150	31.5	326	68.5			1	

^1^The five relevant functional and dysfunctional coping strategies with their respective odds ratios and the corresponding confidence intervals are illustrated. The prevalence rates and the absolute frequencies of the relationship between coping strategy and burnout ‘yes’ and ‘no’ are also shown; *significant/**highly significant.

### Analysis of possible intercorrelations

Within the more detailed analysis of potential intercorrelations between the coping strategies, the following relationships emerged: *Support with friends* were positively associated with *family support* with *r* = 0.198 (*p* < .01) and with *support from fellow students* with *r* = 0.297 (*p* < .01). The coping strategy *support from family* had some correlations, for example, with *support from fellow students* with *r* = 0.212 (*p* < .01). In addition, *family support* is negatively associated with the *use of tranquillisers* (*r* = −0.114; *p* < .01), *use of stimulants* (*r* = −0.176; *p* < .01) and *drinking alcohol* (*r* = −0.145; *p* < .01). The dysfunctional coping strategy *taking tranquillisers* were highly associated with the *use of stimulants* with *r* = 0.410 (*p* < .01) and *drinking alcohol* with *r* = 0.266 (*p* < .01). *Taking stimulants* is also correlated with *drinking alcohol* with *r* = 0.255 (*p* < .01) and furthermore with *withdrawal and ruminating* (*r* = 0.114, *p* < .01). Finally, *withdrawal and ruminating* is associated with *drinking alcohol* with *r* = 0.106 (*p* < .01).

### Results of binary logistic regression

The binary logistic regression confirmed the following functional and dysfunctional coping strategies as highly significantly negative or positive associated with burnout. S*eeking support from friends* (exp(B) = 0.273; *p* < .01), *doing relaxing exercise* (exp(B) = 0.452; *p* < .01), and *doing sports* (exp(B) = 0.610; *p* ≈ .051) were negatively associated with burnout. By contrast, *playing games on the PC or mobile phone* (exp(B) = 2.147; *p* < .01), *withdrawal and ruminating* (exp(B) = 1.776; *p* < .01) and *drinking alcohol* (exp(B) = 1.936; *p* < .01) were positively associated with burnout. The functional coping strategies *seeking support from family members* (exp(B) = 0.635; *p* = .057), and the dysfunctional coping strategies *taking tranquilizers* (exp(B) = 1.900; *p* = .070) and *taking stimulants* (exp(B) = 1.524; *p* = .072) were only marginally significant associated with burnout. According to the regression model *including fellow students* tends to have less impact on burnout than expected. A detailed list of the respective interrelations between the coping strategies is illustrated in supplementary material 2 and the detailed results of the respective effects of all 25 functional and dysfunctional coping strategies on the burnout of the dependent variables are presented in supplementary material 3.

## DISCUSSION

To the best of our knowledge, this is the first study investigating the role of functional and dysfunctional behavioural coping strategies in association to burnout in medical students. The results show high burnout rates among medical students in the 3^rd^, 6^th^, and 9^th^ semesters and among final year medical students, averaging 35%. Current evidence shows varying trends during medical school for the dimension of burnout. Some studies have found that burnout increases with each year of study among medical students [35–38]. Other studies have reported an n-shaped curve with burnout climaxes in the middle phases of the medical curriculum [39–41]. The results of the study presented here indicate that the different semesters show similar prevalence rates for burnout. Looking at the individual dimensions, a heterogeneous picture emerges: Students in earlier stages of university education show lower values for cynicism, but higher values for emotional exhaustion than students in higher stages. Concerning academic efficacy, there was a trend towards less-efficient perception among students in higher education.

A comparison of the results with the reference sample from Gumz and colleagues [22] shows that the medical students examined in this study are more affected by burnout symptoms. Nonetheless, effect sizes are quite small, making the overall result difficult to interpret. It is important to note that in the student cohort of the validation study by Gumz and colleagues, 20% were medical students, others questioned were studying linguistics, natural sciences, economics or law. Gumz and colleagues did not differentiate their results on burnout stress according to specialisation of study, so that it may be that certain specialisations moderate the present result more than other specialisations.

Regarding the study’s main question, highly relevant functional coping strategies were identified, namely, *social support*, more precisely by *friends, family members* and *fellow students, relaxing exercise*s and *sports*. The behavioural-based functional coping strategies found here suggest that social support seems to be a very important method for medical students to reduce the probability of suffering from stress and exhaustion. About 74–93 per cent of the students surveyed said they were actively seeking social support. The detailed results of the intercorrelations showed that there were some moderate intercorrelations between support from family, friends and fellow students. Students seeking family support for stress and burnout were also more likely to consult friends and fellow students. However, binary logistic regression showed that both friends and family had a significant impact on burnout. Correlations between support from friends and fellow students could be explained by the fact that the questionnaire did not distinguish more precisely between intra-university and extra-university friends and that fellow students often represent the friends of students.

Furthermore, medical students explicitly chose sports and relaxation techniques as helpful active stress management strategies. Students who engage in sports or relaxation exercises during stress and exhaustion had a lower probability of suffering from burnout symptoms than students who did not apply these strategies. There is sufficient evidence from the literature that stress-prevention and stress management programmes conducted in groups of medical students have led to a significant reduction of stress and other symptoms for students of human medicine [42–44]. These include different treatments such as relaxation techniques that teach breathing control or muscle relaxation, as well as yoga and meditation. In addition, there are cognitive techniques to reduce and restructure dysfunctional beliefs or thoughts. Very popular and well-known are also approaches for Mindfulness-Based Stress Reduction training (MBSR) that aim to lower reactivity to stress through non-judgmental self-awareness, particularly of physical sensations, cognitions and emotions [45,46]. However, the questionnaire did not differentiate between specific techniques used by medical students. To date, there is no clear evidence from literature which technique provides the greatest potential of stress reduction for medical students and physicians [47,48], but it would have been interesting to know which program or technique students in this study use for stress and exhaustion.

The results may raise concerns on the identified dysfunctional coping strategies *taking tranquilizers, taking stimulants, drinking alcohol, withdrawal* and *ruminating*, and *playing games on PC or mobile phone*. These dysfunctional coping strategies are positively associated with burnout. Using these strategies increases the probability that students will suffer from burnout. In the present study, nearly 50 per cent of the students stated that they retreated and escaped when under stress and exhaustion. Even more alarming is the fact that over 10% of the students surveyed said they were taking drugs for stress and exhaustion and more than 15% were drinking alcohol to reduce stress. The results of the intercorrelations show that the use of alcohol was positively related to the use of drugs and *withdrawal* and *ruminating*, while the use of *tranquilizers* and stimulants also correlated positively. Logistic regression identified especially the use of alcohol, withdrawal and ruminating, as well as computer games as highly affecting burnout. The results of the regression showed that the use of tranquilizers and stimulants was marginally significantly associated with burnout. Some of the students who drank alcohol were more likely to take drugs and to withdraw, so they drank and consumed more by themselves. The finding that social support from the family rather has a protective effect is consistent with this. Students who chose this functional coping strategy preferred less dysfunctional strategies such as alcohol and drugs. Alcohol and drug abuse among practicing physicians [49,50] and medical students [51–53] is supported by literature. Up to 20% of medical students have reported excessive alcohol consumption [54–56]. The use of illicit drugs by medical students is similar to that of age-related peers, and studies indicate that students who use these drugs had started using them before they entered medical school [57,52,58]. Pickard and colleagues [53] found a higher risk of alcohol consumption among Leeds medical students than among the age-matched population. 53% of men and 51% of women regularly exceeded the recommended amount of alcohol per week, while in a sample population in the 18–24 age group only 41% exceeded the safe limit indicated by the WHO. All in all, the risk of substance abuse correlates significantly with the psychosocial impairments experienced, including study stress and working pressure, and was used to reduce tension and cope with loneliness, stress, anxiety and depression [59,60].

## Strengths and limitations

This study investigates the relevance of functional and dysfunctional coping strategies with experienced burnout in medical students. One of the strengths of the study is its large study population of 597 medical students from all stages of university education. This study identified clinically relevant burnout prevalence rates in the investigated groups and analysed an association and potential impact of different coping strategies on burnout in medical students.

One limitation of the study is the cross-sectional design. Consequently, the causal relationship between coping strategies and burnout can be exploratively suggested by the statistical methods used, but not sufficiently interpreted. Therefore, an associative relationship between coping strategies and burnout has to be assumed and the effects of coping strategies on burnout have to be interpreted accordingly. A future longitudinal study with similar cohorts is necessary to further investigate the actual causal relationships.

Students from the 4^th^, 8^th^, and 12^th^ semesters close to their state exams were not interviewed to prevent burnout inflation rates. However, this can lead to an underestimation of burnout prevalence for medical students. Thus, they may not be significantly more burdened than students from other disciplines in the reference study group of Gumz and colleagues [22].

This study measures burnout and potential coping strategies using self-assessment instruments inherently susceptible to distortion. Mixed method designs could be used to present comprehensive as well as individual activity and experience in more detail and to make possible programs sounder.

## Conclusion

The identified functional coping strategies social support of family and friends, relaxation exercises and sports were positive and action-oriented and negatively associated with burnout. The dysfunctional coping strategies such as alcohol and drug use, withdrawal and ruminating and increased gambling on mobile phones and PCs are positively associated with burnout and can be suggested as rather ineffective to deal with burnout. Programmes to improve resilient behaviour and to communicate the identified functional coping strategies are recommended.

## Note

1. The MBI-SS originally developed from the MBI-General Version and originally had 16 items. Through factor analysis and suggestions from the literature, the item ‘When I’m in class or I’m studying I don’t want to be bothered’ was deleted from the dimension cynicism [8].
