# Supplementary material for: Behaviour-based functional and dysfunctional strategies of medical students to cope with burnout
Source: Med Educ Online. 2018 Oct 29;23(1):1535738. doi: 10.1080/10872981.2018.1535738 (PMC6211255; doi:10.1080/10872981.2018.1535738)
Supplement: Supplemental Material [file ZMEO_A_1535738_SM4072.zip › SuppMat/supplementary material_1.docx]

Results of post hoc tests for all dimensions and subgroups.

| **Dimension** | **group (I)** | **group (II)** | **z^1^** | **p^2^** |
| --- | --- | --- | --- | --- |
| **Emotional Exhaustion (EE)** | 3^rd^ semester  3^rd^ semester  3^rd^ semester  6^th^ semester  6^th^ semester  9^th^ semester | 6^th^ semester  9^th^ semester  final year  9^th^ semester  final year  final year | -1.45  -2.37  -2.79  -.809  -1.38  -.501 | >.05  <.05*  <.01**  >.05  >.05  >.05 |
| **Cynicism (CY)** | 3^rd^ semester  3^rd^ semester  3^rd^ semester  6^th^ semester  6^th^ semester  9^th^ semester | 6^th^ semester  9^th^ semester  final year  9^th^ semester  final year  final year | -2.13  -4.95  -4.68  -2.57  -2.32  -.411 | <.05*  <.01**  <.01**  <.01**  <.01**  >.05 |
| **Academic Efficacy (AE)** | 3^rd^ semester  3^rd^ semester  3^rd^ semester  6^th^ semester  6^th^ semester  9^th^ semester | 6^th^ semester  9^th^ semester  final year  9^th^ semester  final year  final year | -2.31  -2.72  -.345  -2.31  -.699  -1.52 | >.05  <.01**  >.05  <.01**  >.05  >.05 |

^1^ z-value within Post hoc tests with Mann–Whitney U; ^2^ corrected with Bonferroni; *significant/**highly significant
