# Supplementary material for: Behaviour-based functional and dysfunctional strategies of medical students to cope with burnout
Source: Med Educ Online. 2018 Oct 29;23(1):1535738. doi: 10.1080/10872981.2018.1535738 (PMC6211255; doi:10.1080/10872981.2018.1535738)
Supplement: Supplemental Material [file ZMEO_A_1535738_SM4072.zip › SuppMat/supplementary material_2.docx]

|  | Functional coping strategies | | | | | Dysfunctional coping strategies | | | | |
| --- | --- | --- | --- | --- | --- | --- | --- | --- | --- | --- |
|  | 1. seeking support from friends | 2. seeking support from family | 3. doing relaxing exercise | 4. doing sports | 5. seeking support from fellow students | 1. taking tranquilizers | 2. taking stimulants | 3. drinking alcohol | 4. ruminating | 5. playing games on PC or mobile phone |
| 1. seeking support from friends |  | r_1_=.198**  p<.01 | n.s.^2^ | n.s. | r=.297**  p<.01 | n.s. | n.s. | n.s. | n.s. | n.s. |
| 2. seeking support from family |  |  | n.s. | n.s. | r=.212**  p<.01 | r= -.114**  p<.01 | r= -.176**  p<.01 | r= -.145**  p<.01 | n.s. | n.s. |
| 3. doing relaxing exercise |  |  |  | n.s. | n.s. | n.s. | n.s. | n.s. | n.s. | n.s. |
| 4. doing sports |  |  |  |  | n.s. | n.s. | n.s. | n.s. | n.s. | n.s. |
| 5. seeking support from fellow students |  |  |  |  |  | n.s. | n.s. | n.s. | n.s. | n.s. |
| 1. 1. taking tranquilizers |  |  |  |  |  |  | r=.410**  p<.01 | r=.266**  p<.01 | n.s. | n.s. |
| 2. taking stimulants |  |  |  |  |  |  |  | r=.255**  p<.01 | r=.114**  p<.01 | n.s. |
| 1. 3. drinking alcohol |  |  |  |  |  |  |  |  | r=.106**  p<.01 | n.s. |
| 4. ruminating |  |  |  |  |  |  |  |  |  | n.s. |

^1^ correlation using kendall tau; ^2^ not significant; ** significant
