# Supplementary material for: Behaviour-based functional and dysfunctional strategies of medical students to cope with burnout
Source: Med Educ Online. 2018 Oct 29;23(1):1535738. doi: 10.1080/10872981.2018.1535738 (PMC6211255; doi:10.1080/10872981.2018.1535738)
Supplement: Supplemental Material [file ZMEO_A_1535738_SM4072.zip › SuppMat/supplementary material_3.docx]

| Coping strategy | exp(B)^1^ | p |
| --- | --- | --- |
| **…seeking support from friends** | **0.273** | **.002** |
| **…doing sports** | **0.610** | **.051** |
| …having a rest | 0.683 | .177 |
| …reading a book | 1.170 | .474 |
| …cooking or baking | 0.742 | .184 |
| …relationship to god or praying | 1.524 | .272 |
| …seeking support from fellow students | 0.782 | .313 |
| **…seeking support from family members** | **0.635** | **.057** |
| …visiting theater or cultural events | 1.130 | .654 |
| …practicing music | 0.690 | .264 |
| **…doing active relaxation exercises** | **0.452** | **.002** |
| …seeking support from parents | 1.653 | .268 |
| …enjoying nature or going for a walk | 1.031 | .899 |
| seeking support from partner | 1.046 | .839 |
| …eating more | 0.936 | .772 |
| **…taking stimulant drugs** | **1.524** | **.072** |
| …surfing in the internet | 0.825 | .425 |
| **…taking tranquilizer** | **1.900** | **.070** |
| **…playing games on the PC or mobile phone** | **2.147** | **.004** |
| …eating less | 1.020 | .941 |
| **… withdrawal and ruminating** | **1.776** | **.009** |
| …smoking cigarettes | 0.991 | .982 |
| **…drinking alcohol** | **1.936** | **.042** |
| …buying useless things | 1.147 | .555 |

^1^ exp(B) represent the logit coefficients as odd ratios, which have been logarithmised. A value of 1 means no change, thus no influence of the predictor. Values less than 1 mean a negative impact on the predictor on the dependent variable (burnout) and values greater than 1 mean a positive influence on the dependent variable (burnout). **Bold** represent (marginal) significant impact of dependent variable (burnout)
